# Current Perspectives and Potential of Probiotics to Limit Foodborne *Campylobacter* in Poultry

**DOI:** 10.3389/fmicb.2020.583429

**Published:** 2020-12-22

**Authors:** Wenjun Deng, Dana K. Dittoe, Hilary O. Pavilidis, William E. Chaney, Yichao Yang, Steven C. Ricke

**Affiliations:** ^1^Center of Food Safety, Department of Food Science, University of Arkansas, Fayetteville, AR, United States; ^2^Diamond V, Cedar Rapids, IA, United States; ^3^Department of Poultry Science, University of Arkansas, Fayetteville, AR, United States; ^4^Meat Science and Animal Biologics Discovery Program, Department of Animal and Dairy Sciences, University of Wisconsin, Madison, WI, United States

**Keywords:** probiotics, *Campylobacter*, poultry, gastrointestinal tract, foodborne pathogens

## Abstract

Poultry has been one of the major contributors of *Campylobacter* related human foodborne illness. Numerous interventions have been applied to limit *Campylobacter* colonization in poultry at the farm level, but other strategies are under investigation to achieve more efficient control. Probiotics are viable microbial cultures that can establish in the gastrointestinal tract (GIT) of the host animal and elicit health and nutrition benefits. In addition, the early establishment of probiotics in the GIT can serve as a barrier to foodborne pathogen colonization. Thus, probiotics are a potential feed additive for reducing and eliminating the colonization of *Campylobacter* in the GIT of poultry. Screening probiotic candidates is laborious and time-consuming, requiring several tests and validations both *in vitro* and *in vivo*. The selected probiotic candidate should possess the desired physiological characteristics and anti-*Campylobacter* effects. Probiotics that limit *Campylobacter* colonization in the GIT rely on different mechanistic strategies such as competitive exclusion, antagonism, and immunomodulation. Although numerous research efforts have been made, the application of *Campylobacter* limiting probiotics used in poultry remains somewhat elusive. This review summarizes current research progress on identifying and developing probiotics against *Campylobacter* and presenting possible directions for future research efforts.

## Introduction

With the introduction of selective media that could be routinely employed for isolation, *Campylobacter* was identified as a critical clinical pathogen associated with the gastrointestinal tract (GIT; On, 2001; Butzler, 2004). By the mid to late 1980s, *Campylobacter* had been recognized as one of the most common bacterial agents causing gastroenteritis worldwide (Allos, 2001; Domingues et al., 2012; Geissler et al., 2017). Currently, *Campylobacter* is considered one of the leading causative agents of bacterial foodborne GIT disease globally (Silva et al., 2011; Mughini-Gras et al., 2014; Kaakoush et al., 2015; Marder et al., 2018), with poultry products being one of the main vehicles of *Campylobacter* exposure (Skarp et al., 2016; Taylor et al., 2013).

In 2010, foodborne transmission accounted for approximately 80 and 76% of campylobacteriosis cases in the United States and in the European Union (EU), respectively (Hald et al., 2016). According to the United States Centers for Disease Control and Prevention (2018), poultry contributed to 33 cases of the 209 foodborne *Campylobacter* outbreaks from 2010 to 2015. Similarly, in the EU, broiler meat and products contributed 24.2% of total foodborne campylobacteriosis outbreaks in 2017 (European Food Safety Authority, 2018). Certainly, preventative measures must be taken in order to reduce the incidence of *Campylobacter* among poultry and poultry products.

**FIGURE 1:**
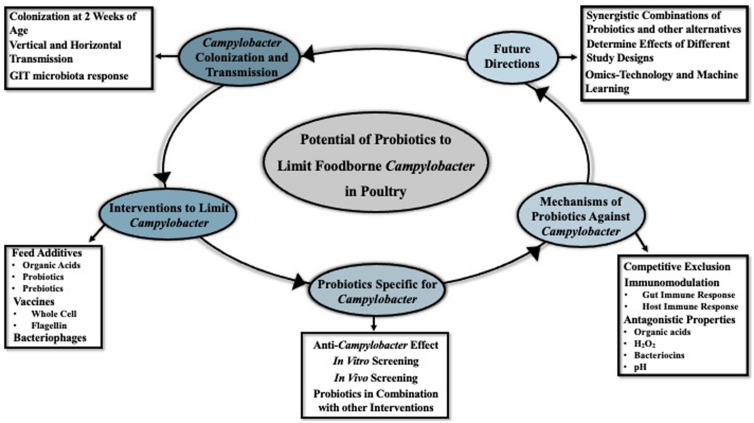
Brief overview of the topics and future directions that are discussed in the current review.

Previously the control measures of *Campylobacter* in poultry included antibiotic treatment, phage therapy, competitive exclusion, and vaccination (Lin, 2009; Meunier et al., 2017; Umaraw et al., 2017). However, the use of antibiotics in livestock can cause a selection of antibiotic-resistant pathogens which further transmit to humans during food consumption, leading to more severe illnesses because of the difficulties in treatment (Gupta et al., 2004; Iovine and Blaser, 2004; Yang et al., 2019). Due to the threat to public health, the use of antibiotics in poultry production has become more restricted (Tang et al., 2017; CDC, 2019). Consequently, in recent years alternative strategies and feed additives to effectively control the colonization of *Campylobacter* in poultry GIT have become of increasing interest (Park et al., 2016; Hossain et al., 2017). This review provides a historical perspective and recent updates on the development of anti-*Campylobacter* probiotics, the effect of host-microbiota on probiotics, and possible directions for future probiotic development research efforts (Figure 1).

## *Campylobacter* Colonization and Transmission in Poultry

### *Campylobacter* Colonization in Poultry

The two most common species, *Campylobacter jejuni*, and *C. coli* are thermophilic and obligate microaerophilic bacteria that colonize the intestinal mucosa of most warm-blood animals, including humans (Newell and Fearnley, 2003). *Campylobacter* spp. prefer to colonize avian species such as wild birds, broilers, turkeys, and ducks (Newell and Fearnley, 2003). The microaerophilic environment and internal body temperature of 41°C in the avian GIT provide optimal environmental conditions for *Campylobacter* (Robyn et al., 2015). The colonization of *Campylobacter* in chickens primarily occurs in the ceca and small intestine. It can still become invasive, appearing in the liver, spleen, deep muscle, thymus, bursa of Fabricius, and blood (Awad et al., 2018). It was reported that once ingested, *C. jejuni* colonization begins in the ileum, followed by dissemination to the jejunum and cecum (Lacharme-Lora et al., 2017). *Campylobacter* rapidly establishes in the chicken ceca and multiplies, eventually reaching very high cecal concentrations (10^9^ CFU/g cecal content; Newell and Fearnley, 2003). Beery et al. (1988) determined the concentration of *C. jejuni* in different GIT locations of 8-day-old chickens, which were orally inoculated with 5 × 10^8^ CFU bacteria cells. The concentrations in the proximal and distal small intestines and the large intestines reached 10^5^ CFU/g and became undetectable at 5 days post-inoculation (minimum level of detection 10^2^ CFU/g). The majority of the colonization occurred in the cecum, which attained a peak of 10^7^ CFU/g 1 day after inoculation and remained detectable even at 7 days. Smith and Berrang (2006) compared the prevalence and concentration of food pathogens between crop and gizzard content in broiler carcasses. The *Campylobacter* prevalence in the crop contents (29 of 29 chickens) was higher than in gizzard contents (12 of 30 chickens). Moreover, it was found that the crops (4.6 log_10_ CFU/mL) contained significantly higher concentrations of *Campylobacter* than the gizzard (2.2 log_10_ CFU/mL).

The colonization of *Campylobacter* in chickens varies due to host age, bacterial strain type, and infective dose (Sahin et al., 2003). Newly hatched chickens are generally free of *Campylobacter* until 1–2 weeks of age; this delay of colonization is referred to as the lag phase (van Gerwe et al., 2009; Pielsticker et al., 2012; Kalupahana et al., 2013). The primary explanation for the lag phase is the protective effect of maternal antibodies (MAB), but the mechanisms behind it have not clearly been described yet (Sahin et al., 2003; Ringoir et al., 2007). The MAB levels are highest in newly hatched chickens but decrease gradually to the background level at approximately 3 weeks of age (Sahin et al., 2003; Shoaf-Sweeney et al., 2008). One study compared the colonization of *C. jejuni* S3B in 3-day-old chickens with and without anti-*Campylobacter* MAB, which were hatched from bacteria-infected and uninfected hens, respectively. When challenged with *C. jejuni* at the concentration of 5 × 10^5^ CFU/bird, the MAB+ chickens exhibited a significantly lower percentage of shedding than MAB- birds at 2 and 4 post-inoculation days (PIDs). However, at 12 PIDs, both groups reached a 100% shedding rate (Sahin et al., 2003). These results indicated the partial protective effect of *Campylobacter* MAB against colonization in young chickens.

Furthermore, Cawthraw and Newell (2010) reported that there was no simple linear relationship between the level of MAB in chicken and their resistance to a *C. jejuni* challenge. It was noted that the resistance of 8-day-old chickens was greater than that for the day-of-hatch birds, although the maternally derived anti-*C. jejuni* IgY serum antibodies were at the peak levels (approximate ELISA OD450 2.1) in the latter one. From 8 to 21 days, the *C. jejuni* resistance of chickens and the antibody levels both decreased. Overall, the 1 to 2-week old birds were more resistant to *C. jejuni* than the 3 week old birds (Cawthraw and Newell, 2010). Another study reported that at least 5 × 10^4^ and 5 × 10^3^ of cells of a *C. jejuni* laboratory-maintained strain were required to colonize 2 and 14-day old chickens, respectively (Ringoir et al., 2007). Ringoir et al. (2007) demonstrated that 2-day old chickens were less susceptible to *C. jejuni* than 14-day chickens. Sahin et al. (2003) reported no interference between high levels of maternal antibodies and the development of the humoral immune system in young chickens. Furthermore, the 21-day-old chickens showed a much higher and more rapid humoral response than the 3-day-old chickens.

Previously *Campylobacter* was considered a non-pathogenic commensal in the poultry intestine, but this concept has been questioned more recently (Humphrey et al., 2014; Awad et al., 2018; Connerton et al., 2018). Humphrey et al. (2014) exposed four commercial broiler chicken breeds to *C. jejuni* M1, and initially, all four breeds produced inflammatory signals in the innate immune response to bacterial colonization. At 12 DPI, three breeds exhibited reduced inflammation responses. They remained asymptomatic after the expression of interleukin-10 (IL-10), while the other faster-growing breed failed to produce IL-10, which led to prolonged inflammation and diarrhea. In addition, Awad et al. (2015) reported changes in intestinal permeability and histomorphology, including decreased crypt depth, villi height, and surface area in *Campylobacter* colonized chickens.

### *Campylobacter* Transmission in Poultry Flocks

*Campylobacter* can rapidly transmit from a colonized individual chicken to the entire flock in a matter of days (Awad et al., 2018). A study conducted with an Australian broiler flock quantified the transmission rate for each *C. jejuni*-infected bird, resulting in estimates of 2.37 ± 0.295 new bird infections per day. Based on this transmission rate, a flock consisting of 20,000 broilers exhibits a 95% prevalence of *Campylobacter* within 4.4 to 7.2 days after the appearance of the first infected bird (van Gerwe et al., 2009). The prevalence of *Campylobacter* in broiler chickens varies depending on the location and can range anywhere between 3 and 90% (Marotta et al., 2015). The transmission routes at the farm are considered to originate mainly from the surrounding environment and are transmitted horizontally between flock mates (Cox et al., 2012; Sibanda et al., 2018).

The vertical transmission (i.e., hen to the egg then to the chick) of *Campylobacter* remains debatable since the observations of vertical transmission have not been consistent across different studies (Cox et al., 2002; Callicott et al., 2006). In fact, Battersby et al. (2016) investigated the transmission of *Campylobacter* on three broiler farms (two flocks per farm) where they took fecal and environmental swabs and used polymerase chain reaction (PCR) assays to detect *Campylobacter*. Battersby et al. (2016) concluded that vertical transmission did not occur but rather the surrounding environment was one of the primary sources of *Campylobacter* and that biosecurity should be considered as a control measure. They concluded that once *Campylobacter* is spread among the flock, it is not long after that *Campylobacter* is detected outside the broiler house. In contrast, Rossi et al. (2012) determined that *Campylobacter* transmission can be due to vertical transmission from the breeder hen. Rossi et al. (2012) inoculated breeder hens intraesophageally and specific pathogenic free (SPF) eggs with *Campylobacter coli.* Rossi et al. (2012) demonstrated the transmission from inoculated hen to egg (offspring) and the potential dangers of *C. coli* reaching the amnion of SPF eggs. However, Rossi et al. (2012) contended that *C. coli* did not appear viable in the infected eggs produced by the inoculated hen, a potential limitation of *C. coli* transmission in the field.

A recent survey on United States broiler production revealed that none of the investigated farm managers (*n* = 18) reported *Campylobacter* tests in their farms, while 11% of poultry veterinarians (*n* = 2) and 90% of processing plants (*n* = 18) included *Campylobacter* in their microbiological tests (Hwang and Singer, 2020). Moreover, only 33% (*n* = 6) of the farms implemented the validated measurements for limiting *Campylobacter* contamination. In the survey, biosecurity was ranked as the most effective strategy against *Campylobacter* on the farm. Still, over half of the farmers and veterinarians reported that biosecurity is not adequate at limiting *Campylobacter* transmission and subsequent infection. These responses collectively indicated that while biosecurity might be the best available choice, it alone may not be sufficiently effective. Thus, further interventions must be utilized to mitigate *Campylobacter* prevalence among poultry, such as dietary supplements that alter *Campylobacter* colonization within the GIT.

### The GIT Microbiota in Poultry and the Impact of *Campylobacter* Colonization

The microbiota of the poultry GIT plays several important roles that benefit host health through the competitive exclusion of pathogens and other non-indigenous microorganisms, stimulation and development of the host immune system, and absorption of nutrients (Shang et al., 2018). It has been determined that most of the bacteria in ileum and ceca of broiler chickens are Gram-positive with low G + C content, which mainly includes *Lactobacillus*, clostridia, *Bacillus*, and *Streptococci* (Lu et al., 2003).

However, the bacterial taxonomic composition of the ceca is significantly different from the jejunum and ileum, which may be attributable to the different functions of these two GIT regions (Gong et al., 2002; Stanley et al., 2014; Awad et al., 2016; Feye et al., 2020). The primary role of the jejunum and ileum is nutrient absorption. However, the ceca serve as the primary site for bacterial fermentation, further nutrient absorption, detoxification, and prevention of pathogen colonization (Gong et al., 2007). In addition, Oakley and Kogut (2016) determined that the fecal and cecal microbiota compositions also differ from one another. At 1-week of age, *Gallibacterium* and *Lactobacillus* were dominant in the feces, while *Bacteroides* was more abundant in the ceca. Both *Clostridium* and *Caloramator* increased significantly in the ceca, whereas *Lactobacillus* remained dominant in the feces of broilers at 6 weeks of age.

In addition to the location of GIT, the composition of microbiota varies depending on the age of broiler chickens. Awad et al. (2016) identified 24 phyla from the GIT contents of 1 to 28-day old broiler chickens. It was revealed that the *Firmicutes* and *Proteobacteria* were the most abundant phyla in all birds. In particular, the number of *Proteobacteria* were found to be significantly higher in newly hatched chickens and subsequently decreased with age, while the *Firmicutes* were predominant in older birds. This microbial community transition appeared to be related to oxygen availability. The facultative anaerobes of the *Proteobacteria* phylum initially colonized the GIT, but as oxygen gradually became depleted, the obligate anaerobes from *Firmicutes* emerged as the dominant microorganisms (Wise and Siragusa, 2007). Oakley et al. (2014) noted that age was more of a driving factor in the diversity and population of the cecal microbiota than dietary treatments. When the birds were 7-day-old, the cecal microbiota were primarily comprised of *Flavonifractor*, *Pseudoflavonifractor*, and *Lachnospiracea*. However, by 21-day of age, *Faecalibacterium* dominated the microbiota of broilers regardless of dietary treatment (Oakley et al., 2014).

Older birds (14–28 days old) exhibited significantly more microbial richness and diversity than young birds (1–7 days old) based on several diversity indices, which included the number of operational taxonomic units (OTUs), Chao1, abundance-based coverage estimator (ACE), Shannon’s index and Simpson index (*p* < 0.01; Awad et al., 2016). Oakley et al. (2014) and Oakley and Kogut (2016) also noted the increase in microbiota richness and diversity as birds matured.

Although some recent research has indicated that *Campylobacter* colonization does not influence the subsequent microbiota composition of commercial broiler flocks (Oakley et al., 2018), significant efforts have been made to determine the influence that *Campylobacter* has on the GIT microbiota. Discussion on the potential interactions between the microbiota and *Campylobacter* in the host GIT has occurred in more recent years (Indikova et al., 2015). Oakley et al. (2013) demonstrated that all samples along the “farm to fork” continuum had a common core microbiota consisting of recognized pathogens such as *Clostridium*, *Campylobacter*, and *Shigella*. Also, Oakley et al. (2013) revealed that *Campylobacter* did not appear to have a significant association with other taxa, concluding that this may be a reason as to why competitive exclusion is not effective against *Campylobacter* (2013). However, *C. jejuni* did have a significant association with *Megamonas hypermegale* (Oakley et al., 2013). Other significant research has revealed that the *C. jejuni* concentrations are higher in the cecal contents of antibiotic-treated or germ-free raised chickens than birds with a conventional microbiota, indicating a host protective role of microbiota against *C. jejuni* (Han et al., 2017).

Sakaridis et al. (2018) randomly sampled 100 birds from four different commercial farms to determine the association of *Campylobacter* loads and the subsequent microbiota composition using 16S rDNA. They determined that the inter-farm variation had a more pronounced effect on the microbiota than the intra-farm variation (Sakaridis et al., 2018). There was no correlation between *Campylobacter* load and the levels of *Firmicutes*, *Bacteroidetes*, or *Ternicutes*; however, there were increased levels of *Enterobacteriaceae* and decreased levels of *Lactobacillus* in the cecal microbiota of birds that had high levels of *Campylobacter* (Sakaridis et al., 2018). Therefore, Sakaridis et al. (2018) suggests that *Lactobacillus* and *Enterobacteriaceae* may be populations to modulate in order to decrease *Campylobacter* colonization.

In addition, Connerton et al. (2018) demonstrated that *Campylobacter* colonized chickens possess a different cecal microbiota composition than those not infected at 2 days post-inoculation. However, as time progressed, age was a more significant driver of the microbiota composition. Upon inoculation, *Campylobacter* colonization of the ceca was followed by a reduction of *Lactobacillaceae* and *Clostridium* cluster XIVa (Connerton et al., 2018). In contrast, Sofka et al. (2015), who compared the microbiota composition between *C. jejuni* positive and negative chickens in the fecal samples, saw no differences in the fecal microbiota composition. However, Sofka et al. (2015) noted that higher proportions of common GIT bacteria such as *Firmicutes* (62 versus 36.6%), *Proteobacteria* (44.6 versus 21.3%), and *Bacteroidetes* (15.4 versus 6.5%) were found among the non-inoculated birds (*Campylobacter* free).

Understanding the colonization of *Campylobacter* in the GIT of poultry and how the residential microbiota may impact or enhance *Campylobacter* infection in poultry enables stakeholders and the industry to combat this rather arduous foodborne pathogen. With this knowledge, researchers have investigated the use of multiple interventions, whether at the preharvest or postharvest level.

### Current and Potential Interventions for Limiting *Campylobacter* in Poultry Flocks

Interventions against *Campylobacter* can be employed at each step of the poultry production chain (i.e., farm, transport, slaughter, processing, and retail; Sahin et al., 2015; Umaraw et al., 2017; Upadhyay et al., 2019). The choice of control measures at each primary poultry production step plays a vital role against *Campylobacter* and could affect the following steps along the food chain (Meunier et al., 2016). Interventions applied at the farm level aim to reduce or eliminate the colonization of *Campylobacter* in broilers, while the remaining steps at postharvest primarily focus on decontamination during processing at the plant (FSAI Ireland, 2002). Due to the focus of the current review, only interventions at the farm level will be discussed. The interventions used at the poultry farm can be broadly categorized as biosecurity interventions, feed additives, vaccines, and bacteriophage (Hermans et al., 2011).

There is a wide range of feed additives, including organic acids such as SCFA, medium-chain fatty acids (MCFA) and their monoglycerides, plant-derived compounds, probiotics, prebiotics, and bacteriocins that are available as potential interventions (Guyard-Nicodème et al., 2016; Meunier et al., 2016; Dittoe et al., 2018; Kim et al., 2019; Micciche et al., 2019). The reported organic and fatty acids include but are not limited to caprylic acid, butyrate, formic acid, and sorbate (Dittoe et al., 2018). The SCFA exerts antibacterial effects by diffusing across the bacterial membrane as non-ionized acids, and dissociating in bacteria cells, which further leads to the decrease of intracellular pH and the dissipation of the proton motive force, and impacting cellular physiology (Ricke, 2003; Sun and O’Riordan, 2013). As reviewed by Meunier et al. (2016), the effectiveness of organic and fatty acids against *Campylobacter* varied widely among studies, and the reproducibility across experiments was low. A study of Guyard-Nicodème et al. (2016) compared the anti-*Campylobacter* effect of 12 feed additives (i.e., monoglyceride, SCFA, plant extracts, probiotics, and a prebiotic-like compound) in broiler chickens over the 42-day sampling period. At 14 days of age, eight additives elicited a significant reduction of *C. jejuni*, while at 35 days, only three additives remained effective, namely, a monoglyceride, a SCFA, and a multi-species probiotic. At 42 days of age, the SCFA still led to more than 2 log_10_ CFU/g reduction of *C. jejuni*, as well as a probiotic and a prebiotic-like compound, which only showed effectiveness at 42 days.

As a result, out of the array of feed amendments available to poultry producers to mitigate *Campylobacter* among their flocks, probiotics are promising. Probiotics, as will be discussed, are capable of directly competing for nutrients against pathogens, excluding pathogens from binding sites, expressing antagonistic mechanisms, and stimulating immunomodulation. A more detailed discussion on probiotics and bacteriocin formation is included in a later section of the current review.

## Probiotics Specific for *Campylobacter* – Identification and Optimization Strategies

Probiotics are considered non-pathogenic and non-toxic viable microorganisms that incur favorable impacts on host health when administrated via an oral route (Lutful Kabir, 2009). Probiotics can be bacteria or yeast and consist of either individual strains or a mixture of several organisms. The more common probiotics are generally comprised of species and strains of *Lactobacillus*, *Streptococcus*, *Bacillus*, *Escherichia coli*, *Bifidobacterium*, and *Saccharomyces*, among others (Dobson et al., 2012; Kergourlay et al., 2012; Helmy et al., 2017; Thibodeau et al., 2017; Massacci et al., 2019). The beneficial effects of probiotics in poultry production include maintaining an optimal balance of GIT microbiota, inhibition of pathogens (Bhatia et al., 1989; Santini et al., 2010; Ritzi et al., 2014), immunomodulation (Cox and Dalloul, 2015), positive histomorphological changes of the ileum (Olnood et al., 2015; Forte et al., 2018), and improving broiler growth performance among others (Kabir et al., 2004). The screening and selection process are critical to ensure that probiotic strains survive the GIT and play their beneficial roles in the animal host. The probiotic strain(s) should be able to survive under GIT conditions (e.g., low pH and bile salt), feed processing and storage conditions (e.g., heat, dry, and starvation), retain high viability and exhibit beneficial effects once reaching the target region of the GIT (Santini et al., 2010; Song et al., 2014; Park et al., 2016).

### The Anti-*Campylobacter* Effect of Probiotics in Chickens

When used as a feed additive, probiotics help to mediate poultry GIT health and reduce the colonization of food and poultry pathogens in the chicken GIT (Cean et al., 2015; Eeckhaut et al., 2016). The addition of a butyrate-producing probiotic strain of *Butyricicoccus pullicaecorum* into a commercial chicken diet significantly reduced the percentage of necrotic lesions caused by *Clostridium perfringens* in all tested trials. It also reduced *Campylobacter* populations in cecal contents by 1.5 log_10_ gene copies/g at day 40 (Eeckhaut et al., 2016). More recently, several studies have screened specific probiotic strains targeted against *Campylobacter* in chickens (Kobierecka et al., 2016a, b; Wang et al., 2019). However, there were variations in reported results among different studies due to the experimental conditions, detection methods, intra- and inter-flock differences of the chicken host, and the complexity of probiotics and host interactions. In turn, these complex interactions led to difficulties in validating the subsequent effects of administering specific probiotic strains (Saint-Cyr et al., 2016; Johnson et al., 2017).

Taha-Abdelaziz et al. (2019) reported the inhibitory effects of six *Lactobacilli* spp. against *C. jejuni in vitro*. Both the neutralized cell-free supernatant and the *Lactobacilli* spp. cell culture inhibited *C. jejuni* growth with clear inhibition zones on Mueller-Hinton (MH) agar. Further investigation found that *C. jejuni* exposed to all *Lactobacilli* spp. except *Lactobacillus reuteri* exhibited a downregulation of genes responsible for motility and invasion, as well as reduced quorum sensing molecule AI-2 production. Another study by Mortada et al. (2020) tested a commercial probiotic for the anti-*Campylobacter* effect *in vitro* and *in vivo*. The overnight cultured gentamicin-resistant *C. coli* was co-cultured at different ratios (1:0, 1:1, 1:5 or 1:10) with the cell-free supernatant of four probiotic strains namely *Lactobacillus reuteri*, *Pediococcus acidilactici*, *Bifidobacterium animalis*, and *Enterococcus faecium* from the commercial product. It was found that the four strains were able to reduce *C. coli in vitro* at a 1:1 ratio or higher. However, when the probiotic product was added to the feed of Cobb-500 broilers, the probiotic supplementation was unable to reduce the colonization of the *C. coli* group at 42 days of age.

The commonly used probiotic strains belong to lactic acid bacteria (LAB), bifidobacteria, *Bacillus* spp., *Bacteroides* spp., and *Streptococcus* spp. (Applegate et al., 2010). Based on the regulation of the United States Food and Drug Administration (US Food and Drug Administration, 2018), the probiotic candidate strains should be generally regarded as safe (GRAS). The probiotic candidate strains usually originate from poultry feces and GIT contents, human feces, cheese, and plant silages (Santini et al., 2010; Ghareeb et al., 2012; Nishiyama et al., 2014; Kobierecka et al., 2017; Smialek et al., 2018). The selection of one or several optimally performing probiotic strains from a broader set of probiotic bacterial candidates is an intensive process. Therefore, tests need to be simple, rapid, and comprehensive (Taheri et al., 2009). The screening process can be roughly divided into two steps: pre-selection *in vitro* and evaluation *in vivo* (Lutful Kabir, 2009). Many screening studies are only carried out *in vitro*, while others are conducted as both *in vitro* and *in vivo* studies. The latter of the screening methods is more informative than the former in determining a probiotic’s candidacy.

### The Screening Procedure of Probiotics for Poultry *in vitro*

*In vitro* screening methods can vary among studies. However, they generally include aggregation and co-aggregation, antibacterial activity, enzymatic activity, cell surface hydrophobicity, survival (acid and bile salt), strain identification and antibiotic sensitivity tests (Tables 1, 2; Taheri et al., 2009; Blajman et al., 2015; García-Hernández et al., 2016). These tests help determine if the probiotic strains would survive when exposed to the extreme conditions of the host GIT environment while still exerting their beneficial functions. The antibacterial test is essential for accurately screening anti-*Campylobacter* probiotics. The cell-free supernatants of probiotics are usually tested using a well-diffusion assay on agar plates to observe the range of anti-*Campylobacter* clearing zones in a *Campylobacter* lawn background. Live cultures of probiotic strains can be tested by methods such as agar spot, agar slab, and as co-cultures in suspension or with GIT cell monolayers (Robyn et al., 2012; Saint-Cyr et al., 2016; Kobierecka et al., 2017; Dec et al., 2018). The *in vitro* co-culture with intestinal cells mimics the interactions between probiotics and pathogens in the host GIT. Šikiæ Pogaèar et al. (2020) tested the *Lactobacillus* spp. strains (*Lactobacillus plantarum* PCS20, PCS22, PCS25, PCK9, and *Lactobacillus rhamnosus* LGG) for their competitive adhesion and infection prevention ability against *C. jejuni* in chicken B1OXI and pig PSI cl.1 epithelial polarized cells. Specifically, in the PSI cl.1 cell line, all tested bacterial strains significantly reduced the adhesion and infection of *C. jejuni* at 3, 17, and 24 h post-infection. In the B1OXI cell line, the bacterial strains PCS22, LGG, and PCK9 significantly reduced the adhesion of *C. jejuni* at 24 h. However, the invasion of *C. jejuni* in the B1OXI cell line was only observed at 3 h post-infection. At that time, the addition of PCS20, PCS22, and PCK9 significantly reduced the invasion of *C. jejuni*, whereas bacterial strains PCS25 and LGG prevented *C. jejuni* invasion.

**TABLE 1:** Summary of tests in screening probiotics *in vitro*.

Tests	Methods	Purpose	References
Aggregation	Probiotic cells clump, gravitate to the bottom of tube and leave a clear supernatant	Related to adhesion ability to epithelial cells	Reniero et al., 1992
Co-aggregation	Ability of probiotic cells aggregate with pathogens	Defense the pathogen colonization	Kmet et al., 1995; Jin et al., 1996; Nami et al., 2019
Anti-bacterial activity	Agar spot test, agar slab assay, well diffusion assay	Anti-*Campylobacter* effect	Schillinger and Lücke, 1989
	Co-culture of probiotic and *Campylobacter* with mammal cell monolayers	Anti-*Campylobacter* effect, Inhibition effects on *Campylobacter* adhesion and invasion	Tabashsum et al., 2018
Cell surface hydrophobicity	Measures decrease of absorbance in cell suspension with added hydrocarbon	Related to colonization and adhesion ability	Rosenberg et al., 1980; Kmet and Lucchini, 1997; Vinderola and Reinheimer, 2003
Bile salts tolerance	Observe cell growth in media containing bile salt	GIT condition	Garriga et al., 1998; Ehrmann et al., 2002
Acid tolerance	Observe cell growth at pH of 2 or even lower	GIT condition	Taheri et al., 2009
Antibiotic susceptibility	Diffusion tests in agar plates	Should not carry antibiotic resistant genes	Bauer et al., 1966; CLSI, 2018

The aggregation and adhesion ability of probiotic bacterial strains facilitates their establishment in the GIT and the exclusion of pathogens (Lebeer et al., 2008). Tareb et al. (2013) investigated the auto-aggregation and co-aggregation ability of viable and heat-inactivated cultures of *L. rhamnosus* CNCM-I-3698 and *L. farciminis* CNCM-I-3699. Both living and dead cells of the two strains showed strong co-aggregation ability with *C. jejuni* CIP 70.2T, which was through the carbohydrate-lectin interaction and proteinaceous components. When probiotic strains were added to mucin at the same time or after *C. jejuni*, the inactivated probiotic cells were more effective than the living cells at preventing *C. jejuni* colonization. The authors indicated that the enhanced adhesion might be because of the production of EPS during the heat inactivation. One advantage of heat-inactivated cells over living cells is enhanced storage stability (Ostad et al., 2009; Ishikawa et al., 2010).

**TABLE 2:** Summary of *in vitro* screenings and methodologies of various probiotic strains against *Campylobacter* in Poultry.

Probiotic(s)	Probiotic Origin(s)	Pathogen	Methods	Reduction	Mechanisms	References
*Lactobacillus salivarius 9b L. salivarius 60d L. johnsonii 8f L. crispatus 49b L. ingluviei 9e L. ingluviei 43d L. oris 50c*	Chicken feces and cloacae	*C. jejuni C. coli*	• Agar slab method for living cells • Well diffusion assay for cell-free supernatant	• Living cells: mean inhibition zone was 18.3 ± 4.3 mm for *C. jejuni*, and 16.7 ± 3.7 mm for *C. coli* • Cell-free supernatant: 16.6 ± 0.5 mm for *C. jejuni*, and 16.5 ± 0.5 mm for *C. coli*	• Antagonism both living cells and cell-free supernatant by production of organic acids	Dec et al., 2018
*L. salivarius, L. plantarum, L. crispatus, L. agilis*	Private collection and broiler feces	*C. jejuni* 12, *C. jejuni* 81–176	• Agar spot test for cell-free supernatant with or without pH neutralization	• Cell free supernatant inhibited growth • Neutralized cell free supernatant did not inhibit growth	• Antagonism by production of acids	Kobierecka et al., 2017
*L. plantarum* PCS 20, *Bifidobacterium longum* PCB 133	Cheese, infant feces	*C. jejuni* strains: CIP 70.2^*T*^, LMG 8842 and 221/05	• Agar spot test using living cells • Well diffusion agar assay with pH neutralized cell-free supernatant (NCS)	• Both PCS 20 and PCB 133 living cells showed >2 mm inhibition zone to all three *C. jejuni* strains • PCB 133 NCS: >2 mm inhibition zone to two *C. jejuni* strains • PCS 20 NCS: >2 mm inhibition zone to one strain	• Antagonism • The living cells of probiotics: organic acid and/or protainaceus molecules • The NCS of probiotics: proteinaceus molecules	Santini et al., 2010
*L. salivarius* SMXD51	Chicken ceca	*C. jejuni* C97ANSES640, *C. jejuni* NCTC 11168, C11168, *C. jejuni* 81–176, and 22 isolates from poultry production line	• Agar well diffuse assay (neutralized cell-free supernatant) • Adhesion and invasion (human HT29-MTX and avian LMH monolayers) • The IL-8 and K60 expression in LMH cell monolayer	• >4 mm inhibition zone: 81–167 and other three strains • No inhibition: C97ANSES640 and other five strains • *L. salivarius* did not protect cell lines from *C. jejuni* adhesion; but reduced *C. jejuni* invasion to HT29-MTX cells by 0.5 logs • *L. salivarius* induced 23.83 ± 8.06 and 48.44 ± 16.06-folds increase in IL-8 and K60 in LMH cells	• Bacteriocin effect was strain-dependent C97ANSES640 was resistant to bacteriocin • A combination of different strategies contributed to the reduction of *C. jejuni*	Saint-Cyr et al., 2017
*L. gasseri* SBT2055 (LG2055)	Human feces	*C. jejuni* 81–176	• Agar well diffusion method using cell supernatant • Cell adhesion and invasion assay	• Untreated and heat treated supernatant reduced *C. jejuni*, while neutralized supernatant did not reduce *C. jejuni* • Different ratios of pre-incubation with *L. gasseri* led to *C. jejuni* 2.5–25-folds decrease on adhesion, and 3–100-folds decrease on internalization	• Inhibition factor of *L. gasseri* was a proteinaceous cell surface component, which mediated co-aggregation with *C. jejuni* • Production of lactic acid and other inhibition mechanisms	Nishiyama et al., 2014
*L. casei*^+^*^*mcra*^* (overexpressed *mcra* gene)	Lab strain	*C. jejuni*	• Cell co-culture in suspension • Cell adhesion and invasion assay • Supplemented with peanut flour	• Co-culture: >6.3 log_10_ CFU/mL at 48 h • Cell-free supernatant: >4.8 log_10_ CFU/mL • *C. jejuni* adhesion and evasion efficiency to HD-11 cell was reduced 0.12 and 0.14 log_10_ CFU/mL and to Hela cells reduced 0.12 and 0.28 log_10_ CFU/mL	• Antagonism Production of large amount of conjugated linoleic acid led to inhibition on adhesion and evasion of *C. jejuni*	Tabashsum et al., 2018

The primary purpose of an antibiotic sensitivity test is to prevent the transposition of antibiotic-resistant genes to nearby resident GIT microbiota (Danielsen and Wind, 2003). It was believed that the probiotics should adhere to the poultry GIT mucosa and maintain viability under harsh conditions, which meant that probiotic strains could potentially have direct contact with intestinal microbiota and transfer antibiotic-resistance genes through horizontal transfer (Luangtongkum et al., 2009; Lutful Kabir, 2009; Verraes et al., 2013). If antibiotic-resistant genes are transferred to pathogens such as *Campylobacter*, it could represent a human health hazard (Imperial and Ibana, 2016). *Lactobacillus* spp. have been identified as candidates for anti-*Campylobacter* probiotics in several studies, but many strains of *Lactobacillus* spp. are resistant to certain antibiotics. Ocaña et al. (2006) reported that six *Lactobacillus* species were able to grow under high concentrations of streptomycin, kanamycin, quinolones (norfloxacin and ciprofloxacin), chloramphenicol, cephalosporins (ceftriaxone and ceftazidime), and aztreonam. Similarly, Taheri et al. (2009) reported a probiotic candidate for poultry, *Lactobacillus crispatus*, exhibited resistance to nalidixic acid and neomycin. Overall, the *in vitro* selection process helps exclude unqualified candidate strains and narrows the range of required screening. Ultimately, only a few strains that perform outstandingly well in the *in vitro* tests should be selected for *in vivo* tests.

### The Screening Procedure of Probiotics for Poultry *in vivo*

Although the *in vitro* tests characterized probiotic strains under conditions mimic the intestinal environment, they cannot reproduce the exact interactions that occur among probiotics, the host GIT microbiota, and the possible GIT immune response (Table 3; Saint-Cyr et al., 2016, 2017; Mortada et al., 2020). The selected candidate strains that were inhibitory *in vitro* may not elicit a reduction of *Campylobacter in vivo* (Robyn et al., 2012, 2013). Thus, the selected probiotic strains should be further evaluated *in vivo* to determine their colonization ability, anti-pathogen effects, and persistence in chicken GIT (Lutful Kabir, 2009). Blajman et al. (2015) conducted a study to select chicken-originated probiotic strains for feed supplementation. In that study, 360 bacterial strains from broiler chicken GIT contents were screened through a series of *in vitro* tests including aggregation test, antagonistic activity, bacterial identification, cell surface hydrophobicity, acid resistance, bile tolerance, and H_2_O_2_ production tests, and the three best performing probiotic strains were selected for follow up *in vivo* testing. These three strains were constructed to be rifampicin-resistant to track their colonization within the chicken GIT. The administered strain concentrations in the liver, crop, and cecum were determined by direct plating on de Man, Rogasa, and Sharpe agar plates supplemented with rifampicin (MRSrif). In the end, *Lactobacillus salivarius* DSPV001P was selected as the candidate probiotic strain since it successfully colonized and maintained significantly higher population levels in the broiler GIT.

**TABLE 3:** Summary of the *in vivo* screening of probiotic strains against *Campylobacter* in poultry.

Probiotic(s)	Probiotic Origin(s)	Pathogen	Methods	Reduction	Mechanisms	References
*Bifidobacterium longum* PCB 133	Infant feces	*C. jejuni* CIP 70.2^*T*^, LMG 8842 and 221/05	•15–20 days old chickens treated with *B. longum* suspension daily for 15 days	•One log reduction after 15 days administration	Not specified	Santini et al., 2010
*Bacillus subtilis* (enhanced motility)	14-day-old Cobb 500 broiler chicks	*C. jejuni*	•Selected motility enhanced strains fed to chicks daily from day of hatch •At 7 days, challenged with *C. jejuni*	•The motile isolates achieved 1–2 log CFU/g reduction •Least 0.5 log CFU/g more reductions than original probiotic isolates did	Swimming ability of motile strains enable them reach *C. jejuni*. Compete for nutrients, binding sites, and produce antimicrobial compound	Aguiar et al., 2013
*L. gasseri* SBT2055 (LG2055)	Human feces	*C. jejuni* 81–176	•Day old chicks orally inoculated 10^6^ CFU of *C. jejuni* •24 h post-inoculation, *L. gasseri* (10^8^ CFU) fed *ad libitum* in diet •14 days post-inoculation, cecal contents quantified for *C. jejuni*	•About 250-fold decrease at 14 days post-inoculation •Less colonization in mucosal surface	Co-aggregation with *C. jejuni* and other unknown mechanisms	Nishiyama et al., 2014
*L. plantarum* PA18A	Privately owned and commercial chicken stools	*C. jejuni*	•Challenged at 14 days •Tested at 4 and 8 days after infection	•1 log_10_ reduction at 4 days after infection	Did not mention mechanisms, could be lactic acid production	Kobierecka et al., 2017
*L. salivarius* SMXD51	Chicken ceca	*C. jejuni* C97ANSES640	•The *L. salivarius* SMXD51 culture (10^7^ CFU) were orally administered 1 day after hatching then every 2–3 days until 35 days •*C. jejuni* (10^4^ CFU) challenged at day 11 •Immune response evaluated by RT-qPCR: IL-8 and K60	•*C. jejuni* in cecal contents: 0.82 log at 14 days •2.81 log at 35 days •The IL-8 and K60 expression in cecal tonsil significantly increased at 35-day chicken with *L. salivarius*	Reduction not directly through bacteriocin Inhibition of adhesion and/or immune modulation Combination of these three	Saint-Cyr et al., 2017
PoultryStar sol (*Enterococcus faecium, Pediococcus acidilactici, Bifidobacterium animalis, L. salivarius, L. reuteri*)	Multispecies probiotic product	*C. jejuni* 3015/2010	•*C. jejuni* infected chickens orally at day of hatch •At the same day, probiotic product was added to drinking water 2 or 20 mg/bird/day to chickens	•Reduction in cecal colonization: •8 days post-challenge (3.77–5.81 log_10_ CFU/g reduction) •15 days post-challenge (5.5–5.85 log_10_ CFU/g reduction)	Not investigated, likely to be the production of antimicrobial compounds such as organic acids	Ghareeb et al., 2012
Lavipan (multispecies probiotic): *Lactococcus lactis* IBB 500, *Carnobacterium divergens* S-1, *L. casei* OCK 0915, L0915, *L. plantarum* OCK 0862, *Saccharomyces cerevisiae* OCK 0141	Chicken feces, turkey feces, carp gut, plant silage	*Campylobacter* spp. (field study)	•Lavipan supplemented diet fed *ad libitum* •37 days, birds processed •Feces, pectoral muscles and environmental samples were tested for *Campylobacter*	• Feces samples: no reduction (<0.5 log CFU/ml) •Pectoral muscle: no reduction Environmental samples: >1 log CFU/ml reduction	Not specified	Smialek et al., 2018

One way to ensure the colonization of the lower GIT is through encapsulation or microencapsulation of the probiotic. In fact, some probiotic strains with anti-*Campylobacter* properties cannot survive the acidity of the host stomach, so encapsulation is necessary. Arsi et al. (2015a) used intracloacal inoculation of probiotics to introduce them more directly to the lower GIT of birds for *in vivo* screening without the extra cost for encapsulation. They compared intracloacal administration to oral gavage on the anti-*Campylobacter* effects of ten pre-selected probiotic strains. The birds were challenged with *C. jejuni* on day 7, and on day 14, *C. jejuni* cecal concentrations were quantified. Only one strain of oral administered probiotics achieved a 1-log reduction in the ceca, whereas the six strains introduced via intracloacal administration resulted in 1–3 log reductions of *C. jejuni*.

During the *in vivo* screening procedures, additional characteristics enable the probiotic strains to achieve better anti-*Campylobacter* performance in the poultry host. Motility enhancement of probiotic strains was identified as a critical characteristic for the reduction of *Campylobacter* colonization in the GIT. Aguiar et al. (2013) developed a screening technique for selecting probiotic strains with enhanced motility. The *in vivo* experiments indicated that motile strains reduced at least 0.5 log_10_ CFU/g more *C. jejuni* in the cecum than the original strains. The enhanced ability allowed the motile strains to reach the deep mucosal surface of cecal crypts, and overcome *C. jejuni* by occupying the binding sites, competing for nutrients, and/or by the production of antibacterial compounds.

In order to meet the needs of industrial production, the viability and persistence of probiotics under storage conditions such as lyophilization need to be evaluated as a practical consideration following the *in vivo* studies (Blajman et al., 2015). The in-feed stability and viability of probiotics will ensure that a sufficient level is administered to the host to deliver the expected anti-*Campylobacter* effects under production conditions (Ren et al., 2019).

### Probiotics in Combination With Other Interventions to Inhibit *Campylobacter*

Probiotics can also be applied with other *Campylobacter* control measures to improve the effectiveness of the overall intervention. Such combinations that exist are probiotics coupled with vaccines, phytochemicals, and prebiotics. Nothaft et al. (2017) reported that the co-administration of probiotic strains *Anaerosporobacter mobilis* or *L. reuteri* increased the efficacy of a *C. jejuni* vaccine in both broiler and Leghorn layer chickens. This research group developed an *N*-glycan-expressing *Escherichia coli* live vaccine that induced a specific immune response and achieved a several log reduction of *C. jejuni* in the host. However, the vaccine was not protective in some birds where lower numbers of *A. mobilis* were present in the GIT. When co-administered with either *A. mobilis* or *L. reuteri*, a higher proportion of chickens were protected against *C. jejuni* by the vaccine, accompanied by increased body weight and production of antibodies against the *C. jejuni N*-glycan antigen.

In an *in vitro* co-culture conducted by Tabashsum et al. (2019), berry pomace phenolic extracts (BPPE) stimulated the growth and enhanced the anti-*Campylobacter* effects of a conjugated linoleic acid overproducing *Lactobacillus casei* (LC-CLA). The co-culture of the cell-free supernatant from LC-CLA with the presence of BPPE reduced *C. jejuni* over 3.2 log_10_ CFU/ml while BPPE alone or LC-CLA+ BPPE only decreased *C. jejuni* by approximately 1.8 log_10_ CFU/ml (Tabashsum et al., 2019). Also, in the presence of BPPE, the LC-CLA living cell and cell-free supernatant both exhibited a much stronger inhibition against the *C. jejuni* adhesion and invasion of the DF-1, HD-11, and HeLa cell monolayers. For instance, LC-CLA + BPPE reduced both adhesion and invasion of *C. jejuni* to HD-11 by 1 log_10_ CFU/ml. However, in the same research group’s previous study, the reduction by LC-CLA alone reduced adhesion and invasion to HD-11 by approximately only 0.07 and 0.14 log_10_ CFU/ml (Tabashsum et al., 2018, 2019).

Fooks and Gibson (2002) investigated the anti-pathogen effect of probiotic strains *L. plantarum* 0407 and *Bifidobacterium. bifidum* Bb12 that utilized various prebiotics as carbohydrate sources. The probiotics were co-cultured with pathogens in a basal media that was supplemented with different prebiotics [fructooligosaccharide (FOS), inulin, and xylooligosaccharides (XOS)], and their paired mixtures. Regardless of experimental conditions, the probiotic and prebiotic combinations of *L. plantarum* + FOS, *B. bifidum* + FOS, *B. bifidum* + Inulin: FOS (80:20 w/w), and *B. bifidum* + FOS: XOS (50:50 w/w) significantly reduced *C. jejuni* growth in the basal medium compared to the probiotic strains-only groups. The anti-*Campylobacter* effects of probiotic and prebiotic combinations varied depending on the type of prebiotics used. This variation might be because fermentation was affected by the different prebiotic structures, which impacted pathogen inhibition and the end products produced by the probiotics. Arsi et al. (2015b) also attempted to improve the anti-*Campylobacter* efficacy of three probiotic strains (*Bacillus* spp., *L. salivarius* subsp. *salivarius* and subsp. *salicinius*) by the supplementation with prebiotics in broiler chickens. Two prebiotics, FOS (0.125, 0.25, or 0.5% concentration) and mannan oligosaccharide (MOS, 0.04%, 0.08 or 0.16% concentration) were combined with each probiotic strain and fed to the day-of-hatch chickens. The chickens were challenged with *Campylobacter* on day 7, and cecal concentrations were quantified on day 14. The combination of 0.04% MOS and *L. salivarius* subsp. *salicinius* led to a 3-log reduction of *Campylobacter*, whereas the probiotic alone only resulted in a 1 to 2 log reduction. In addition, Baffoni et al. (2017) found that the life-long administration of a synbiotic (probiotic *Bifidobacterium longum* subsp. *longum* PCB133 and prebiotic XOS) effectively protected the chicken host against *Campylobacter* more than a short-term supplementation (starting at 14-day old). Compared to 10-day-old chickens, the plate counts of *Campylobacter* in 39-day-old chickens were reduced by approximately 4.8 and 3.8 log CFU/g with the prolonged and discontinued supplementation of the synbiotic treatment. However, qPCR-based quantification showed no significant reduction of *Campylobacter* between 10 and 39-day old chickens in both treatment groups. Thus, the choice of detection method can contribute to a different conclusion in a study.

Ultimately, the screening procedures, *in vitro* and *in vivo*, allow for the implementation and use of probiotics able to survive and modulate the GIT. Without the ability to survive the GIT, the probiotic would never be able to limit or reduce *Campylobacter* in the hindgut. Further, these methodologies help us better understand how to combine these screened probiotics with other feed amendments. These combinations are the future of feed amendments in poultry as they allow for sustained affects in the GIT. However, it is important to understand the exact mechanisms behind the probiotic in order to combine with other amendments or supplements.

## Functional Mechanisms of Probiotics Against *Campylobacter* in Poultry

Probiotics play multiple roles in the poultry host, delivering beneficial effects, such as increasing nutrient uptake, and body weight gains. The mechanisms behind the host beneficial effects are complex and not always well defined, so only the mechanisms of antibacterial effects are discussed in this review (Lutful Kabir, 2009; Park et al., 2016; Popova, 2017; Wang et al., 2017; Peralta-Sánchez et al., 2019). The current known modes of action for probiotics as an antimicrobial is demonstrated in three ways including, but not limited to, competitive exclusion, antagonism, and stimulation of the host immune system (Zhang et al., 2007; Bratz et al., 2015; Cox and Dalloul, 2015; Schneitz and Hakkinen, 2016; Dec et al., 2018).

### Competitive Exclusion

The competitive exclusion (CE) concept was first developed by Nurmi and Rantala (1973) when they attempted to limit the *Salmonella* proliferation in broiler flocks. Conceptionally, this approach introduced the intestinal or fecal bacteria from healthy *Salmonella*-free adult chickens to newly hatched chicks to reduce the *Salmonella* colonization in these chicks (Lutful Kabir, 2009; Boulianne et al., 2019). Later the CE concept was applied for controlling other enteropathogens, including *Campylobacter*, *Clostridium*, and *E. coli* in poultry production (Stern and Meinersmann, 1989; Stern et al., 2001; La Ragione et al., 2004; Schneitz, 2005). The traditional CE cultures utilize a mixture of undefined bacterial species and populations from the chicken GIT instead of known bacterial species, leading to conflicting observations among studies. Furthermore, the specific mechanisms of the undefined CE cultures were difficult to ascertain (Schoeni and Doyle, 1992; Schoeni and Wong, 1994). Numerous studies have been carried out to derive candidate anti-*Campylobacter* strains from chicken GIT contents and elaborate on the potential mechanisms of CE specifically directed toward *Campylobacter*. In general, the CE characteristic of probiotics acts through the nutrient competition and occupation of mucosal niches to reduce the *Campylobacter* colonization in the poultry host (Mead, 2000; Chaveerach et al., 2004; Pan and Yu, 2014). As reported by Nishiyama et al. (2014), the probiotic strain *Lactobacillus gasseri* SBT2055 (LG2055) reduced up to 25- and 100- fold of *C. jejuni* 81-176 adhesion and internalization to the human epithelial cell monolayer (Int407) *in vitro*. When daily orally administered to chickens, *L. gasseri* reduced *C jejuni* colonization by 250- fold in the cecum of 14-day-old birds post-challenge compared to the levels in the control group. It was found that a proteinaceous component on the LG2055 cell surface resulted in its co-aggregation with *C. jejuni* or competitive adhesion to Int407 cells, indicating that this surface component might play a vital role in the CE activity against *C. jejuni* (Nishiyama et al., 2014). To gain insight into the inhibition mechanism, the same group of researchers carried out another study focusing on the role of cell surface aggregation-promoting factors (APFs) of LG2055 (Nishiyama et al., 2015). The APFs are associated with the self-aggregation, maintenance of cell shape, and adhesion of *L. gasseri*. The study revealed that the primary inhibition mechanism of the AFP mediated competition was through adhesion to epithelial cells, instead of co-aggregation with *C. jejuni*. The LG2055 *apf1* gene deletion mutant lost its inhibition effect to *C. jejuni* on Int407 cell monolayer and in the chicken GIT, while the wild type LG2055 reduced *C. jejuni* invasion *in vitro* by 177- fold and colonization *in vivo* by 230- fold.

Ganan et al. (2013) reported the protective effect of commercial human probiotic strains, *L. rhamnosus* GG, *Propionibacterium freudenreichii* ssp. *shermanii* JS and *Lactococcus lactis* ssp. *lactis*, on the poultry GIT mucus layer against *Campylobacter* infection. The intestinal mucus from broiler and turkeys was isolated and coated on microtiter plate wells for *in vitro* exclusion and competitive inhibition assays. When applied before *Campylobacter* infection, the probiotics reduced *Campylobacter* colonization by occupying the binding sites on intestinal mucus isolated from jejunum, colon, and cecum. However, when the probiotics and *Campylobacter* were simultaneously exposed to the mucus, the probiotics increased *Campylobacter* adhesion to the mucus. *In vitro*, *L. casei* outcompeted the attachment of *C. jejuni* to human epithelial cells INT407 when they were introduced at 1:1 ratio in a co-infection assay (Salaheen et al., 2014). When the ratio of *L. casei* and *C. jejuni* was lowered to 1:10, *L. casei* still significantly reduced *C. jejuni* colonization. However, further decreases in the number of *L. casei* to a 1:100 ratio failed to reduce *C. jejuni* attachment. The authors suggested that the initial number of *L. casei* should be high enough to exclude the *C. jejuni*. Therefore, the inhibition mechanism of *L. casei* may have been through the occupation of the host cell surface receptors that *C. jejuni* uses to recognize and subsequently bind to.

### Antagonism

The antagonistic effects of probiotics include the production of antibacterial metabolites such as organic acids, H_2_O_2_, and bacteriocins. As many probiotic candidates are Lactic Acid Producing Bacteria (LAB), it is common for probiotics to produce a sufficient amount of organic acids to alter the pH of the surrounding environment and reduce pathogens. As such, Chaveerach et al. (2004) reported a probiotic candidate (*Lactobacillus* P93) isolated from the chicken GIT inhibited the growth of *Campylobacter* by producing organic acids and anti-*Campylobacter* proteins. In an *in vitro* co-culture, Neal-McKinney et al. (2012) reported that lactic acids produced by *Lactobacillus* were able to disrupt the cell membranes of *C. jejuni* leading to cell death. Similarly, the *in vitro* screening of the cell-free culture supernatants of seven *Lactobacillus* strains in a well-diffusion agar assay revealed the inhibition of *C. jejuni* and *C. coli* (Bratz et al., 2015). This inhibitory effect was achieved by the production of organic acids, which decreased the pH.

When the supernatants were pH neutralized before testing, they lost the inhibitory effect against *Campylobacter*. Another *in vitro* study also reported the inhibition of *Campylobacter.* Seven *Lactobacillus* isolates from the chicken GIT inhibited the growth of *Campylobacter* by producing organic acids that decreased the surrounding pH (Dec et al., 2018). However, the anti-*Campylobacter* effect was no longer observed after the pH of the cell-free supernatant was neutralized to 6.5 to 7.0. In some cases, the anti-*Campylobacter* ability of probiotics is due to the combined effects of the antagonistic and competitive exclusion properties. For example, in an *in vitro* study, the *Lactobacillus fermentum* 3872 was reported to bind to the same *C. jejuni* attachment receptor in host GIT as well as releasing lactic acid that inhibited *C. jejuni* (Lehri et al., 2017).

To support their invasion and establishment in the host, all major lineages of bacteria and archaea produce bacteriocins, antimicrobial peptides (Gillor et al., 2009; Svetoch and Stern, 2010; Hoang et al., 2011a; Lagha et al., 2017). The antibacterial function of bacteriocins is through the formation of pores in the cell wall. Bacteriocins bind to the cell wall of the target microorganism and interact with the outer cell membrane, leading to the formation of these pores and leakage of ions which, in turn, causes the death of the target cell (Svetoch and Stern, 2010; Prudêncio et al., 2015).

Based on *in vitro* studies, *L. salivarius* has been identified as a promising probiotic candidate due to its well-characterized ability to produce bacteriocins and anti-*Salmonella* and anti-*C. jejuni* effects (Messaoudi et al., 2013). More recently, 44 strains of LAB were screened as potential probiotics by Ayala et al. (2019). Of the 44 screened strains, *L. salivarius* L28 was the “top-ranking” strain and possessed the most antagonistic features such as no antimicrobial resistance (AMR)-encoding genes in mobile elements, ability to produce bacteriocins and adhere to the epithelial cells, and low cytotoxicity percentages. In other studies, *L. salivarius* SMXD51, MMS122, and MMS151 were reported to be ideal bacteriocin producers against *Campylobacter* (Messaoudi et al., 2011). Saint-Cyr et al. (2017) tested the bacteriocin of *L. salivarius* SMXD51 *in vitro* against 23 *C. jejuni* strains isolated from poultry farms and retail operations. The bacteriocin exhibited inhibition (<4 mm inhibition zone) of 15 strains and strong inhibition (>4 mm inhibition zone) of two strains of *C. jejuni* (AC4700 and C94). The remaining four *C. jejuni* strains were not inhibited by the bacteriocin, indicating the difference in sensitivity of the *C. jejuni* strains to the *L. salivarius* SMXD51 bacteriocin.

Thus, the variation of bacteriocin sensitivity among *Campylobacter* strains presents difficulties in applying bacteriocin as an on-farm control measure. Hoang et al. (2011b) tested the prevalence of bacteriocin-resistance in *C. jejuni* and *C. coli* isolates from various sources including humans (15), bovines (5), chickens (121), turkeys (1), pigs (4) and environment [i.e., trapped mice (5), bird droppings (5) and lagoons (1)]. Except for one *C. coli* strain, all the strains were susceptible to the tested bacteriocins, OR-7 and E-760, produced by chicken derived probiotic strains *L. salivarius* and *E. faecium*, respectively. The MIC of *C. coli* was 64 mg/mL, compared to other strains with MIC ranges between 0.25 and 4 mg/mL. To identify the genes involved with bacteriocin resistance, the authors compared the OR-7 resistant *C. jejuni* mutant strain with the parent strain using microarray analysis. It was concluded that the multidrug efflux pump CmeABC played a role in the bacterial resistance to the OR-7 bacteriocin. Previous research had revealed the contribution of *CmeABC* to the resistance against antibiotics and natural antimicrobials in *Campylobacter*.

Although the multidrug efflux pump *CmeABC* is responsible for the resistance of *Campylobacter* against bacteriocins, bacteriocins are still effective against *Campylobacter*. Stern et al. (2008) demonstrated that two cell-free bacteriocins (250 mg/kg feed) produced by *Paenibacillus polymyxa* and *L. salivarius* reduced *C. jejuni* by at least a 6 log_10_ CFU in chickens. In contrast, the ingestion of living cells (10^7^–10^8^ CFU/chick) of the producer strains did not elicit any inhibitory effects toward *C. jejuni*. These two strains failed to exclude the *C. jejuni* and occupy the colonization sites in chicken intestine. The bacteriocin was potentially produced in the host GIT in limited amounts. However, there was no reason for the strain to overproduce bacteriocins, given the high energy costs for carrying plasmid and toxin production (Dobson et al., 2012). Another potential reason for the ineffectiveness of the bacteriocin producers was that their populations were too low compared to the surrounding microorganisms. In this case, the impact of the bacteriocin was limited. The advantage of killing other competitors could not compensate for the metabolic cost for the production of bacteriocin (Riley and Wertz, 2002). Also, the GIT might not present the optimal environmental conditions for triggering the maximum *in vivo* production of bacteriocin, unlike laboratory growth medium and conditions (Telke et al., 2019).

### Immunomodulation

#### The Effect of *Campylobacter* on the Chicken Gut Immune Response

Thus far, few studies have demonstrated a probiotic-triggered immune response against *Campylobacter* in poultry. However, the interaction between *Campylobacter* and the chicken immune system is not well characterized, unlike other food pathogens such as *E. coli* and *Salmonella* (Shaughnessy et al., 2009; Chintoan-Uta, 2016). In chickens, the gut-associated lymphoid tissues (GALTs) play a crucial role in the poultry intestinal immune system. The GALT is comprised of lymphoid structures such as the bursa of Fabricius, cecal tonsils, Peyer’s patch, Meckel’s diverticulum, and lymphocyte aggregates distributed in the epithelial lining and the lamina propria (Lillehoj and Trout, 1996; Kim and Lillehoj, 2019). The poultry immune system of poultry includes the innate and adaptive immune responses, with the latter system further divided into the humoral and cell-mediated immune response. The initiation of the innate immune response begins with the recognition of pathogen-associated molecular patterns (PAMPs) by the pathogen recognition receptors (PRRs; Kim and Lillehoj, 2019). There are several important receptors in chicken GIT responsible for bacteria recognition, including the Toll-like receptor (TLR)-2 for peptidoglycan, TLR-4 for lipopolysaccharides, TLR-5 for flagellin, and TLR-21 for unmethylated CpG DNA of bacteria (Wigley, 2013). The corresponding recognition by these receptors leads to the production of antimicrobial peptides and cytokines (e.g., IL-10) by the epithelial cells, further activating the lymphocytes. In addition, the signal triggers the B cells to produce secretory IgA (sIgA) (Kim and Lillehoj, 2019).

The adaptive immune response is an antigen-specific response by B cells and T cells. The triggered B cell differentiates and produces antigen-specific immunoglobulin (Ig) antibodies that interact and destroy the extracellular antigens (Lillehoj and Trout, 1996; Erf, 2004). However, when antigens have already entered host cells, the cell-mediated response plays a role in eliminating the intracellular antigens (Erf, 2004). The cell-mediated immune response includes the activation of different cells such as T lymphocytes, NK cells, and macrophages, whereas T cells are further divided into cytotoxic T lymphocytes (CD8+) and helper T cells (CD4+) (Lillehoj and Trout, 1996).

The colonization of *Campylobacter* has been reported to trigger both innate and adaptive immune responses of the chicken host. Vaezirad et al. (2017) reported that the immunosuppressed chickens exhibited more rapid *C. jejuni* colonization and dissemination to the liver. At 17 days of age, the chickens were treated with glucocorticoid (GC), which dampened the innate immune response. In that study, higher concentrations of bacteria were present in the cecal contents and liver compared to the non-GC treated group 2–4 days post the *C. jejuni* challenge. These results indicated that the chicken immune system might play a role in limiting the invasion and dissemination of *C. jejuni*. The role of B lymphocytes on the colonization of *C. jejuni* in broilers has also been studied. Lacharme-Lora et al. (2017) compared healthy chickens to B lymphocyte depleted chickens to determine the exact role of B lymphocytes on *C. jejuni* colonization (Lacharme-Lora et al., 2017). At day-of-hatch, chickens were bursectomized using cyclophosphamide. The bursectomy depleted over 90% of bursal B cells and disabled the anti-*C. jejuni* IgY and IgM production under *C. jejuni* exposure. At 14 and 28 days post-inoculation (DPI), the cecal *C. jejuni* levels were high regardless of the bursectomized or control groups. However, *C. jejuni* in the jejunum and ileum cleared by 28 DPI in the control groups and not in the bursectomized group. By 63 DPI, the shedding levels of *C. jejuni* in the control birds decreased while the bursectomized group remained unchanged. Therefore, the B lymphocytes played an essential role in the small intestine but not in the ceca during *C. jejuni* colonization.

#### The Effect of Probiotics on the Chicken Immune Response

The GIT microbiota and probiotics are known to stimulate the immune response against pathogens (Mahfuz et al., 2017; Willson et al., 2018). Several studies have reported the enhancing effects of probiotics on the chicken immune response (Brisbin et al., 2015; Shojadoost et al., 2019; Šefcová et al., 2020). The presence of probiotics trigger the host immune response, depending on the bacterial strains and experimental conditions.

Haghighi et al. (2006) reported elevated production of several antibodies in day-of-hatch chickens fed a probiotic cocktail containing *Lactobacillus acidophilus*, *B. bifidum*, and *Streptococcus faecalis*. Specifically, there were increases of immunoglobulin G (IgG) in the GIT against tetanus toxoid (TT) and IgG and IgM in serum against TT and the *Clostridium perfringens* alpha-toxin, compared to non-probiotic treated chickens. At 6 weeks of age, the supplemented probiotic *Bacillus subtilis* enhanced the serum IgM levels and the cell-mediated immune response in chickens grown under high ambient temperatures (ave. 29–32°C) compared to the negative control group (Fathi et al., 2017). The probiotics showed no significant effect on IgA and IgY levels (Fathi et al., 2017). In contrast, Bai et al. (2017) reported increased levels of IgA and IgG levels in the serum of chickens fed *B. subtilis* fmbJ supplemented diets at 42 days of age.

Furthermore, Sadeghi et al. (2015) reported an enhanced response to the Newcastle and infectious bursal viruses when *Salmonella* challenged broilers were fed diets supplemented with a commercial probiotic (Gallipro, Chr Hansen, Milwalkee, WI, United States). The challenge of *Salmonella enterica* decreased the antibody titer, lymphocyte count, and the weight of spleen and bursa in chickens fed a no-probiotic supplemented diet. However, broilers fed the *B. subtilis* supplemented diets showed increased levels of antibodies against the Newcastle virus (18%) at 21-days of age and against the Newcastle (21%) and infectious bursal viruses (14%) at day 42. Also, the virus antibodies were not affected by the *B. subtilis* probiotic under *Salmonella*-free conditions. Though the antibody increase due to the supplementation of the probiotic diet was not further investigated, the authors offered some potential explanations. First, *B. subtilis* could reduce *Salmonella* colonization through inhibitory mechanisms such as CE and antagonism, thus reduce the negative effect of *Salmonella* on antibody levels. Also, *Salmonella* is known to stimulate the production of interleukin-1β (IL-1β), and *B. subtilis* were reported to suppress the pro-inflammatory cytokines, including IL-1β and increase the anti-inflammatory cytokines. Another more recent *in vitro* study investigated the immune-modulation caused by *L. salivarius*, *L. johnsonii*, *L. reuteri*, *L. crispatus*, and *L. gasseri*. The treatment of chicken macrophages with every species of the heat-killed *Lactobacilli* at a multiplicity of infection (MOI) of 100 increased nitric oxide (NO) production (an indicator of macrophage activation) except for *L. reuteri*. Moreover, the treatment of the heat-killed single or mixed cultures significantly increased the phagocytosis by macrophages on the fluorescein isothiocyanate labeled *C. jejuni* (Taha-Abdelaziz et al., 2019).

## Conclusion and Future Directions

The feed supplementation of probiotics has been reported as an effective pathogen control strategy for poultry over several decades; however, a thorough mechanistic understanding is still missing for it to be utilized as a routine measure to limit *Campylobacter* colonization. In the present review, the characteristics of *Campylobacter* colonization and transmission in poultry were discussed. Also, the current preharvest intervention measures were briefly addressed, followed by a discussion on anti-*Campylobacter* probiotics. The screening process and functioning mechanisms of probiotics were reviewed. The number of studies on probiotics screening is considerable, but the variations among studies led to difficulties for direct comparisons to reach general conclusions. Several studies may observe the reduction of *C. jejuni* by the same probiotic strain, but their reported number of log reductions may differ. This variation might be contributed by the difference of experimental conditions and design, such as the broiler age, the time of administration of the probiotic treatment, and subsequent *Campylobacter* challenge. In addition, the potential functional mechanisms of probiotics were discussed, which may help understand the kinetics of *Campylobacter* reduction and provide directions for future screening work.

Overall, the addition of probiotics to feed is effective for reducing *Campylobacter* in poultry, but it cannot achieve complete elimination. Therefore, more studies on the effects of combining probiotics with other control preventions such as prebiotics and postbiotics represent an opportunity to reduce or eliminate *Campylobacter*. The inconsistency of *Campylobacter* reduction by probiotic treatment is another barrier hindering the widespread application of probiotics at the farm level. The effect on probiotic performance by factors including *Campylobacter* strain, variations among farms, different genetic lines, feed composition, and environmental procedures should be further elaborated. In addition, the laborious and time-consuming drawbacks of probiotic screening work have hindered the progress of the application of probiotics in poultry production. In more recent years, the availability of omics technology (i.e., metagenomic, transcriptomic, and metabolomic methods) has provided researchers a means to estimate the functions of GIT bacteria and their host and their responses to pathogens based on the characterization of the bacterial and host genetic information (Ricke et al., 2019). Thus, the utilization of omics can be employed to efficiently facilitate the identification of probiotic candidates (Papadimitriou et al., 2015; Rebollar et al., 2016). These approaches, combined with machine-learning techniques, have been widely studied for applications in numerous fields, including microbiology. The ability to predict the interactions among microorganisms can be applied in probiotic screening and therefore enable a more rapid screening process (Qu et al., 2019; van den Bogert et al., 2019; Zhou and Gallins, 2019).

## Author Contributions

All authors significantly contributed to the work of the current review. WD wrote the current review with the assistance from DD who formatted, edited, and submitted the review with direction from SR. HP, WC, YY, and SR edited the review before submission.

## Conflict of Interest

HP and WC are employees of Diamond V. The remaining authors declare that the research was conducted in the absence of any commercial or financial relationships that could be construed as a potential conflict of interest.
